# Sequencing Immunotherapy and Hypofractionated Radiotherapy in Frail Patients with Locally Advanced Head and Neck Squamous Cell Carcinoma

**DOI:** 10.3390/curroncol33050239

**Published:** 2026-04-22

**Authors:** Beatrice Bettazzi, Viola Salvestrini, Marco Banini, Olga Ruggieri, Annarita Palomba, Ilaria Camilla Galli, Lorenzo Livi, Pierluigi Bonomo, Carlotta Becherini

**Affiliations:** 1 Department of Experimental and Clinical Biomedical Sciences M. Serio, University of Florence, 50134 Florence, Italy; bettazzibeatrice04@gmail.com (B.B.); lorenzo.livi@unifi.it (L.L.); 2 Department of Radiation Oncology, Careggi University Hospital, 50134 Florence, Italy; viola.salvestrini@unifi.it (V.S.); marco.banini@unifi.it (M.B.); carlotta.becherini@unifi.it (C.B.); 3 Istituto Fiorentino di Cura e Assistenza (IFCA), 50139 Florence, Italy; o.ruggieri@giomi.com; 4 Histopathology and Molecular Diagnostics, Careggi University Hospital, 50134 Florence, Italy; annarita.palomba@aouc.unifi.it (A.P.); galliic@aou-careggi.toscana.it (I.C.G.)

**Keywords:** HNSCC, unfit, elderly, frail, hypofractionated, radiotherapy, sequence, immunotherapy, locoregional control

## Abstract

(1) Background: Hypofractionated radiotherapy and immunotherapy (IT) are possible treatment options for HNSCC patients unsuitable for standard curative treatment, yet no high-level evidence supports their combined use. We aim to report on the clinical outcome of a single-center cohort of HNSCC patients treated with a hypofractionated radiotherapy (hypoRT) regimen in combination with IT alone or chemo-immunotherapy (CT-IT). (2) Methods: We enrolled a cohort of elderly and frail HNSCC patients unsuitable for standard curative treatment, deemed candidates to undergo hypoRT in a sequential strategy (time interval < 6 months), followed or preceded by IT alone (hypoRT_IT) or CT-IT. We selected our sample using the Geriatric 8 (G8) score and the Charlson Comorbidity Index (CCI). (3) Results: At a median follow-up of 11 months (IQR 5–20), the median locoregional control (LRC) was 12 months (95% CI 7.0–17.1) with a 1-year progression-free survival rate of 63%. For the hypoRT-IT group, the median overall survival was 12 months (95% CI 0–24). No grade (G) 4–5 in-field acute side effects were observed, while one case of G3 oral mucositis and two cases of G3 radiation dermatitis were reported. (4) Conclusions: A sequential combination of checkpoint inhibitors and hypoRT may provide clinical benefit with acceptable toxicity in frail and elderly patients with advanced HNSCC unfit for standard therapy.

## Simple Summary

No standard of care exists for elderly and frail patients diagnosed with locally advanced head and neck squamous cell carcinoma (HNSCC). Indeed, many patients may not qualify for standard radiation alone or in combination with platinum-based chemotherapy. In the recurrent/metastatic setting, pembrolizumab alone is a first-line treatment option for elderly or unfit patients for chemo-immunotherapy. Hence, emerging strategies are under investigation to improve the outcome for this growing patient population. We aim to report on the clinical outcome of a prospective cohort of elderly and frail HNSCC patients treated with a hypofractionated radiotherapy regimen in association with immunotherapy alone or chemo-immunotherapy.

## 1. Introduction

Head and neck squamous cell carcinoma (HNSCC) remains a significant challenge in oncology, particularly in elderly and frail patients who may not tolerate standard treatments. The current standard of care for locally advanced HNSCC involves concomitant chemoradiation (CRT) with platinum-based chemotherapy [1,2,3]. However, many elderly or unfit patients may not be eligible for CRT. In this scenario, exclusive radiation therapy (RT) is the standard option, but reported outcomes are not satisfactory [3,4,5]. Moreover, many of these patients may not be suitable candidates for radical RT either, due to factors such as advanced age, comorbidities, poor performance status (PS 2), or significant baseline weight loss (≥10%) [6]. The use of hypofractionated RT (hypoRT) for elderly and frail HNSCC patients has been extensively investigated over the past decade, with multiple studies demonstrating its feasibility and efficacy. Although no single fractionation regimen can be regarded as standard, international guidelines [7] recommend employing hypoRT as an adequate approach to this patient category. The introduction of immune checkpoint inhibitors as a tolerable treatment option for patients with Combined Positive Score (CPS) positive recurrent/metastatic HNSCC raises the question of how to exploit immunotherapy and hypofractionated RT in frail patients with non-metastatic disease.

It is well known that hypofractionation reduces the overall treatment time and the number of hospital visits, which is particularly beneficial for elderly and frail patients with limited mobility [7,8]. Potentially, higher doses per fraction may enhance immunogenic cell death (ICD) and augment the antitumor immune response when combined with IT [9,10,11,12].

As of today, for elderly and frail patients with locally advanced disease, not amenable to standard CRT, there is no data to support the combination of immunotherapy (IT) and RT. Algorithms to support decision-making in this context have been proposed, yet no definitive guidance is available for clinical practice [5,13].

Thus, the aim of our study is to report on the clinical outcome of a small cohort of elderly and frail locally advanced HNSCC patients unfit for standard curative therapy who received hypo RT and IT with or without chemotherapy. Moreover, we complemented our findings by reviewing the literature on the unmet need of non-metastatic HNSCC with low performance status.

## 2. Materials and Methods

### 2.1. Study Design and Patient Population

This prospective cohort study included patients diagnosed with locally advanced HNSCC who were considered unfit for a standard treatment regimen after multidisciplinary team evaluation and treated at our institution between September 2018 and August 2024. All patients in this cohort had a positive CPS score (one group with CPS 1–19, one group with CPS greater than 20) and were unsuitable for standard full- dose RT due to factors such as age, comorbidities, or poor performance status [14,15]. Patients were treated according to protocol, consisting of hypoRT followed sequentially (<6 months window) by either IT or CT-IT [16,17]. The decision to employ a sequential rather than concurrent strategy was informed by emerging evidence suggesting that concurrent administration of IT and RT may result in increased radiation-induced lymphopenia and immunosuppression [11]. Based on these results, immunotherapy was initiated either before RT or within 2–4 weeks after RT completion, avoiding strict concurrent administration [16,17].

### 2.2. Patient Selection and Geriatric Assessment

Geriatric 8 (G8) questionnaire and Charlson Comorbidity Index (CCI), in addition to Eastern Cooperative Oncology Group Performance Status (ECOG PS), were used to define frailty in our cohort [14,15]. G8 score is based on a scale of 0 to 17, where a higher score indicates a better health status, and the cut-off score of 14 or less (≤14) is considered a sign of frailty [14,18]. The Charlson Comorbidity Index is composed of the following parameters: age, myocardial infarction, congestive heart failure, peripheral vascular disease, cerebrovascular accident or transient ischemic attack, dementia, chronic pulmonary disease, connective tissue disease, peptic ulcer disease, liver disease, diabetes mellitus, hemiplegia, moderate to severe chronic kidney disease, solid tumor, leukemia, lymphoma and Acquired Immune Deficiency Syndrome [19,20]. Every single condition weighs from 1 to 6 points, with a CCI scale of 0 to 15 [21,22].

### 2.3. Radiotherapy Regimen

We defined hypoRT as treatments with a single fraction of a minimum 2.5 Gy (range 2.5–7 Gy), with an equivalent dose in 2 Gy fractions (EQD2) of 44 Gy and a biological effective dose (BED) of 73.33 Gy [23,24]. HypoRT was administered to gross tumor volumes, omitting or minimizing elective nodal volumes as per clinical judgment, in order to optimize locoregional control while potentially preserving immune function [10]. All patients underwent a simulation CT scan of the head, neck, and chest using a thermoplastic mask for immobilization and were treated either with Volumetric Modulated Arc Therapy (VMAT) or Tomotherapy. The planning goal was to cover 95% of the PTV with 95% of the prescribed dose. Organ at Risk dose constraints were adopted following DAHANCA guidelines [25]. The treatment had to be delivered <6 months from the start of IT or CT-IT.

### 2.4. Immunotherapy and Chemotherapy Regimen

Immunotherapy schemes included IT alone or with concurrent chemotherapy, prescribed as clinically indicated [16,17].

The CT-IT regimen consisted of carboplatin, 5-fluorouracil, and pembrolizumab on day 1 every three weeks. Carboplatin was administered by intravenous infusion at AUC 3 (10 mg/mL), 5-Fluorouracil was administered by continuous infusion at the dose 4000 mg/mq (50 mg/mL) for 96 h, while pembrolizumab was administered by intravenous infusion at a flat dose of 200 mg (25 mg/mL).

Patients CT-naïve and treated with IT alone received either Nivolumab or Pembrolizumab. Nivolumab was administered on day 1 every two weeks by intravenous infusion at a flat dose of 240 mg (10 mg/mL) until unacceptable toxicity or disease progression, while pembrolizumab was administered on day 1 every three weeks by intravenous infusion at a flat dose of 200 mg (25 mg/mL) until unacceptable toxicity or disease progression.

### 2.5. Literature Review Methodology

A comprehensive literature review was conducted using electronic databases including PubMed, Scopus, Web of Science, and Cochrane Library [26,27]. Keywords utilized included “hypofractionated radiotherapy,” “immunotherapy,” “checkpoint inhibitors,” “HNSCC,” “elderly patients,” “platinum-unfit,” “sequential therapy,” and “elderly cancer treatment” [28,29]. The objective was to gather articles addressing the efficacy and safety of combined RT and IT for elderly or frail patients with HNSCC, with particular attention to prospective or retrospective studies exploring innovative approaches for patients ineligible for standard chemoradiotherapy [30].

Sources were selected based on criteria of quality, relevance, and contemporaneity, prioritizing publications from the last 5 years [31,32]. Each study was examined to collect data on demographic characteristics, treatment regimens, side effects, and survival outcomes, with the intent of identifying best practices and gaps in the treatment of the target patient population [33].

### 2.6. Statistical Analysis

Patients’ characteristics and treatment features were collected. Survival outcomes were assessed by the Kaplan–Meier method [34,35]. Kaplan–Meier analyses were performed to estimate overall survival (OS), disease-free survival (DFS), locoregional control (LRC), and locoregional event-free survival (LR-EFS) [36,37]. Stratified analyses were conducted according to PD-L1 CPS categories (>20 vs. 1–19), G8 score (<12 vs. ≥12), and CCI (<5 vs. ≥5) [38,39].

The log-rank test was used to compare survival curves between subgroups [40,41]. Cox proportional hazards regression models were constructed to identify independent prognostic factors for OS, including variables such as age at treatment initiation, number of IT administrations, CPS score, G8 score, CCI, ECOG PS, tumor stage, and treatment response [42,43]. Receiver operating characteristic (ROC) curve analysis was performed to evaluate the prognostic accuracy of significant variables identified in the Cox model, with area under the curve (AUC) values calculated to assess discriminatory ability [44,45].

Statistical significance was set at *p* < 0.05 for all analyses. All statistical analyses were performed using appropriate statistical software [46].

### 2.7. Ethics and Informed Consent

The study was conducted in accordance with international and local ethical regulations [47]. All patients provided written informed consent prior to treatment initiation, with a clear understanding of the risks, benefits, and therapeutic alternatives [48]. The study obtained approval from the institutional ethics committee [49].

## 3. Results

### 3.1. Patients and Treatment Characteristics

A total of 23 patients were included in the study, with a median age of 81 years (range: 51–88 years). Female and male patients were 12 and 11, respectively. The median Geriatric 8 (G8) score was 10.5 (range: 6–16 points), and 21 patients were considered vulnerable with an individual score ≤ 14. The median Charlson Comorbidity Index (CCI) score was 5 (range: 1–8). The median ECOG PS was 1, but nine patients had an ECOG PS of 2 [Table 1].

Overall, 12 patients had T4 stage disease, and 5 had N2 nodal disease. The oral cavity was the most frequent primary site (12 patients), followed by oropharynx (5 patients), larynx, hypopharynx, and paranasal sinuses. Twelve and 11 patients had CPS ≥ 20 and 1–19, respectively.

The majority of patients were treated with 40 Gy in 16 fractions (16/23 subjects), corresponding to a BED of approximately 56 Gy (α/β = 10) and an EQD2 of 46.7 Gy. Alternative fractionation schedules employed in our cohort included regimens ranging from 2.5 to 7 Gy per fraction, all falling within the biological dose range considered potentially immunogenic (BED 50–116.67 Gy) [46,47]. Fourteen patients were CT-naïve candidates for hypoRT-IT alone: 4 and 10 patients received nivolumab and pembrolizumab, respectively. The median number of IT administrations was 8 (range: 2–32) [Supplementary Table S1]. Two patients reported a complete response. The first patient was a 51 years old caucasian woman, with ECOG PS 2, CCI of 1, G8 score of 7.5, stage T4N3M0 SCC oral cavity cancer. The CPS score was >20. She underwent CT-IT regimen (with consolidation regimen IT), and the RT fractionation scheme was 40 Gy/16#, started a month after the first IT cycle. The treatments were overall well tolerated, with only CT-related G2 anemia and a RT-related G3 acute radiodermatitis, which regressed to G2 after 3 months and then further improved. The patient is still alive and continuing the maintenance IT with pembrolizumab (32 cycles). The second patient was a 75 years old caucasian man, with ECOG PS 1, CCI of 7, G8 score of 12.5, diagnosed with T4N3aM0 SCC oropharynx cancer. The CPS score was >20. He underwent IT with pembrolizumab alone with a consolidation regimen, and the RT scheme was 40 Gy/16#, started 3 months after IT. No G ≥ 3 adverse events were reported, either for IT or RT. The patient underwent a total of 18 cycles of IT until disease progression. The patient died 4 months after discontinuing treatment. 

### 3.2. Clinical Outcomes

**Figure 1:**
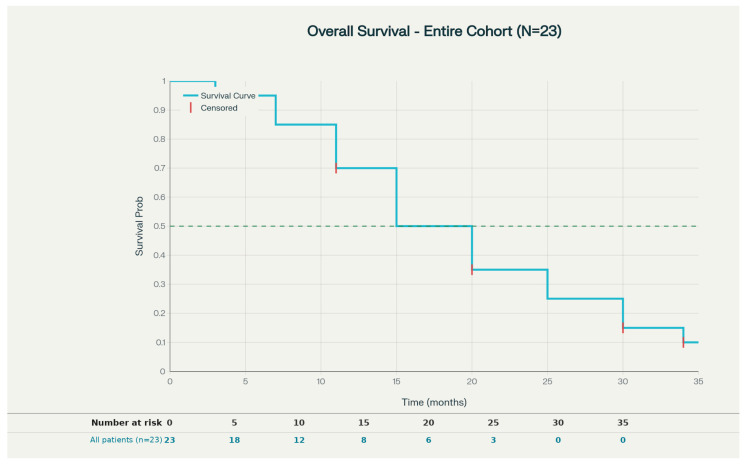
At a median follow-up of 15.1 months (range: 3–34 months), the median overall survival (OS) for the entire cohort was 15 months (95% CI, 11.0–19.2).

At a median follow-up of 15.1 months (range: 3–34 months), the median overall survival (OS) for the entire cohort was 15 months (95% CI, 11.0–19.2) [Figure 1]. The 1-year OS rate was 61%. For the hypoRT-IT group specifically, the median OS was 12 months (95% CI, 0–24). Seven patients (30%) were alive at the end of follow-up. The median disease-free survival (DFS) was not reached, with a mean DFS of 24.8 months (95% CI, 20.9–28.6). The median locoregional control (LRC) was 12 months (95% CI, 7.0–17.1), with a 1-year progression-free survival (PFS) rate of 63%. The median locoregional event-free survival (LR-EFS) was 20 months (95% CI, 4–20), with a mean of 15.4 months (95% CI, 10.9–19.9). Notably, the best response to immunotherapy showed a statistically significant impact on LRC (*p* = 0.0361), while the best response to RT did not demonstrate a significant impact.

Stratified Kaplan–Meier analysis revealed several important trends, even though not reach statistical significance. Patients with CPS > 20 (group 1) demonstrated a numerically longer median OS of 26 months compared to 15 months for CPS 1–19 (group 2) (*p* = 0.9256). For DFS, the median was not reached for CPS >20 patients, compared to 18 months for CPS 1–19 patients (*p* = 0.5151). For LR-EFS, patients with CPS > 20 showed a mean of 19.0 months compared to 8.5 months for CPS 1–19 (*p* = 0.2204). Patients with G8 ≥12 (less frail) had a median OS of 20 months compared to 12 months for G8 < 12 (*p* = 0.4376). The mean DFS was similar between groups: 24.7 months (G8 ≥12) vs. 23.0 months (G8 < 12, *p* = 0.4376). Patients with CCI < 5 (fewer comorbidities) demonstrated a median OS of 20 months compared to 12 months for CCI ≥ 5 (*p* = 0.2928). Notably, for DFS, patients with CCI < 5 showed a median of 28 months compared to 18 months for CCI ≥ 5, with a trend toward statistical significance (*p* = 0.0559). Additionally, explorative analyses are shown in the Supplementary Materials.

In terms of toxicity, no grade (G) 4–5 in-field acute side effects were observed in our cohort. Three cases of G3 acute toxicities were reported: one case of G3 oral mucositis and two cases of G3 radiation dermatitis.

## 4. Discussion

The use of hypoRT for elderly and frail HNSCC patients has been extensively investigated over the past decade, with multiple studies demonstrating its feasibility and efficacy (Table 2). The ELAN-RT trial, a multicenter randomized non-inferiority study, compared split-course hypoRT (55 Gy/20 fractions) with standard fractionation (70 Gy/35 fractions) in frail elderly patients ≥ 70 years [50]. The trial demonstrated that hypofractionation was associated with lower toxicity and fewer treatment interruptions, making it a suitable option for this vulnerable population. 

The recent phase III HYPNO trial, conducted in 10 low- and middle-income countries with 792 patients, demonstrated non-inferiority of hypoRT (55 Gy/20 fractions over 4 weeks) compared to normofractionated accelerated RT (66 Gy/33 fractions over 5.5 weeks), both with optional weekly cisplatin [51]. At 3 years, locoregional control was 50.7% vs. 51.2% (*p* = 0.36), and grade 3+ late adverse events were 18.8% vs. 20.2% (*p* = 0.68), confirming non-inferiority for both endpoints [51]. This trial provides strong evidence supporting hypofractionation as a resource- sparing alternative for locally advanced HNSCC [51].

Several studies have explored various hypofractionated regimens specifically for elderly and frail patients. Mohanti et al. reported on short-course palliative RT with 20 Gy in 5 fractions, demonstrating effective symptom palliation with acceptable toxicity [52], a dinding further confirmed in a randomized controlled trial [53]. The “quad shot” regimen, consisting of 14.8 Gy delivered in four twice-daily fractions over 2 consecutive days, has emerged as a popular palliative option. Ghoshal et al. demonstrated that this regimen provided symptom relief in all patients, with a median OS of 5.7 months and no grade 3–4 toxicities [54]. Corry et al. reported objective responses in 84.6% of patients treated with quad shot, with minimal toxicity and positive impact on quality of life [55].

Systematic reviews and meta-analyses have demonstrated that IT is as effective and safe in older patients as in younger patients. Salvestrini et al. conducted a systematic review showing that elderly patients with R/M HNSCC derived similar OS benefit from immunotherapy compared to younger patients, with a manageable toxicity profile [56]. This finding is consistent with data from other tumor types, where immune checkpoint inhibitors have demonstrated age-independent efficacy [57]. Only sparse evidence is available to support the sequential combination of IT and hypoRT in non-metastatic patients not amenable to receive standard curatively intended treatment due to their general conditions. 

Several studies have specifically addressed IT in elderly HNSCC patients [13,56,57]. They suggested that those with negative PD-L1 at diagnosis may benefit from induction hypoRT to upregulate PD-L1 expression for improved immune response [58]. Zandberg et al. conducted a randomized phase II trial comparing sequential vs. concurrent pembrolizumab with chemoradiotherapy in patients with locally advanced HNSCC [16]. At 4-year follow-up, the sequential arm (pembrolizumab starting 2 weeks after CRT) demonstrated superior outcomes compared to the concurrent arm: locoregional control 96% vs. 64% (HR 0.11, *p* = 0.012), PFS 69% vs. 49% (HR 0.55, *p* = 0.132), and OS 83% vs. 71% (HR 0.51, *p* = 0.17) [16]. A significant increase in macrophages, PD-L1+ macrophages, and PD-L1+ tumor cells was noted with treatment in the concurrent but not the sequential arm, reflecting immunosuppressive changes in the tumor microenvironment [16].

Moreover, the palliative “quad shot” regimen is being explored with ICIs and has yielded durable responses in small HNSCC series [58,59,60]. The combination was well tolerated, with 23% of patients experiencing grade 3 toxicities overall [60]. The median OS of 9.4 months compared favorably to historical controls of 4.5–5.7 months with quad shot alone [54,55].

Our cohort study adds to the available body of evidence, demonstrating that a sequential combination of hypoRT and IT may provide clinical benefit with acceptable toxicity in frail and elderly patients with non-metastatic HNSCC who are deemed unfit for standard curative treatment. The median OS of 15 months in this heavily pretreated, frail population (median age of 81 years, 91% with G8 ≤ 14) compares favorably with historical controls and demonstrates the potential of this approach in a challenging patient population.

Our median OS of 15 months in an unfit, elderly population compares favorably with outcomes from standard palliative approaches. Historical studies of palliative quad shot radiotherapy alone reported median OS of 4.5–5.7 months [54,55]. Recent studies combining quad shot with IT have reported median OS of 9–10 months [60], still shorter than our results. This suggests that our sequential approach, which includes both a moderately hypofractionated RT regimen and extended immunotherapy, may offer superior outcomes compared to either modality alone or very short- course palliative approaches.

With the stratified analysis employed in the present study, we were able to provide insights into the potential utility of biomarkers for patient selection. While none of the stratifications (CPS, G8, CCI) reached statistical significance for OS, likely due to our small sample size, important trends emerged that warrant discussion. Significant numerical differences were found in OS according to G8 and in both OS and DFS according to CCI. These findings are consistent with previous studies demonstrating that G8 score is a significant predictor of outcomes in elderly cancer patients [14,18] and that comorbidity burden may be an important factor in patient selection for this treatment approach [19,20]. Based on the purely exploratory analyses from our work, inherently limited by the small sample size, sequencing IT with hypoRT may be prioritized in patients with CPS > 20, CCI < 5, and those predicted to tolerate extended IT cycles. Chronological age alone should not be a contraindication; rather, comprehensive geriatric assessment using validated tools (G8, CCI) should guide selection [18].

Close monitoring for both efficacy (clinical and radiographic response) and toxicity is essential, with a low threshold for dose modification or treatment interruption given the vulnerable patient population [61].

The favorable toxicity profile observed in our study—no grade 4–5 acute toxicities and only 13% grade 3 toxicities—represents a major advantage over standard concurrent CTRT, which typically results in 40–60% grade 3–4 acute toxicities in elderly patients [3,4]. This is particularly important in the elderly and frail population, where treatment-related toxicity can result in hospitalization, treatment interruption, and deterioration in performance status that may never fully recover [6].

The low toxicity profile observed in our study is consistent with previous reports of hypo-RT in elderly HNSCC patients. Bonomo et al. reported that a similar 40 Gy in 16 fraction schedule resulted in grade 3 acute side effects in 36% of patients, with no grade 4–5 toxicities [62]. Similarly, Porceddu et al. demonstrated that 40 Gy in 10 fractions resulted in no grade 4 mucositis or dermatitis, with effective symptom palliation [63].

**Table 2:** Summary of key studies in the literature review. Abbreviations: RCT, randomized controlled trial; RT, radiotherapy; IT, immunotherapy; LRC, locoregional control.

Study (Year) [Ref.]	Study Type	N	RT Regimen	IT Combination	Key Finding
HYPNO trial (2023) [51]	Phase III RCT	792	55 Gy/20 fr vs. 66 Gy/33 fr	None (+/− cisplatin)	Non-inferiority: 3 y LRC 50.7% vs. 51.2%
Porceddu et al. (2007) [63]	Prospective	46	40 Gy/10 fr	None	82.6% primary response, 84.7% nodal
Quad Shot series [54,55]	Multiple cohorts	110+	40 Gy/16 fr or 20 Gy/5 fr	None	Good symptom control, low toxicity
Hughes et al. (2023) [58]	Pilot trial	15	Quad-shot (4 × 3.7 Gy)	+ pembrolizumab	Promising durable responses

Quality of life preservation is paramount in elderly cancer patients, particularly those with limited life expectancy due to age and comorbidities. By reducing the number of treatment visits (16 vs. 33–35 for standard fractionation), minimizing acute toxicity, and avoiding the morbidity of concurrent chemotherapy, our approach addresses a critical unmet need in this population [6,63]. Future studies should include formal quality-of-life assessments to quantify this benefit [64].

Several limitations of our study warrant acknowledgment. First, the sample size of 23 patients limits statistical power to detect differences between subgroups. The near-significant finding for CCI stratification of DFS (*p* = 0.0559) suggests that a larger study might reveal additional significant associations. Second, the heterogeneity in radiotherapy fractionation schedules (range 2.5–7 Gy per fraction), while all falling within the hypofractionated and biologically effective range, introduces variability that may obscure dose–response relationships. Third, the lack of a concurrent control group limits causal inference, though ethical considerations make randomization of frail elderly patients to intensive standard treatment problematic. Fourth, our follow-up period (median 15.1 months) is relatively short for assessing late toxicity, which typically manifests beyond 12–24 months. A longer follow-up will be necessary to fully characterize the late toxicity profile of this approach. Fifth, we did not perform detailed immune profiling or serial biopsies to characterize the tumor microenvironment changes induced by our sequential treatment, limiting our ability to validate the mechanistic hypotheses underlying our approach.

Future research directions should include prospective randomized trials, biomarker studies, mechanistic studies, optimization of RT fractionation, combination with novel immunotherapy agents, and integration of proton therapy.

## 5. Conclusions

Our report of 23 patients supports that a sequential combination of hypofractionated radiotherapy and immunotherapy may provide clinical benefit with acceptable toxicity in frail elderly patients with locally advanced HNSCC deemed unsuitable for standard curative treatment.

## Figures and Tables

**Table 1:** Patients characteristics.

Features	Median Value/Number (Range)
*Age*	81 (55–88)
*Gender*	
Female	12
Male	11
*ECOG PS*	1 (0–2)
*CCI*	5 (1–8)
*G8*	10.5 (6–16)
*Tumor site*	
Oropharynx	5
Oral cavity	12
Paranasal sinuses	2
Hypopharynx	2
Larynx	2
*TNM Stage*	
*T*	
T2	2
T3	3
T4	12
*N*	
N1	5
N2	5
N3	1
*CPS*	
0	0
1–20	11
>20	12

Abbreviation list. ECOG PS: Eastern Cooperative Oncology Group Performance Status; CCI: Charlson Comorbidity Index, 0–16 points score; G8: Geriatric 8, geriatric screening tool, 0–17 points score; TNM stage: Tumor, Node, and Metastasis staging; CPS: Combined Positive Score, to evaluate the expression of a protein called PD-L1 on tumor cells and immune cells.

## Data Availability

The data presented in this study are available on request from the corresponding author due to privacy restrictions.

## Supplementary Materials

The following supporting information can be downloaded at: https://www.mdpi.com/article/10.3390/curroncol33050239/s1, Table S1: treatment characteristics. Figure S1: Patients with CPS >20 (group 1) demonstrated a numerically longer median OS of 26 months compared to 15 months for CPS 1–19 (group 2), although this difference did not reach statistical significance (*p* = 0.9256). Figure S2: Patients with CCI < 5 (fewer comorbidities) demonstrated a median OS of 20 months compared to 12 months for CCI ≥ 5 (*p* = 0.2928). Figure S3: Abbreviations: IT, immunotherapy; AUC, area under the curve; CPS, Combined Positive Score; G8, Geriatric 8; CCI, Charlson Comorbidity Index; ECOG PS, Eastern Cooperative Oncology Group Performance Status; NS, not significant. Blue lines and dots indicate statistically significant findings (*p* < 0.05). Figure S4: ROC analysis confirmed the prognostic value of both variables: number of immunotherapy administrations (AUC = 0.75) and age at the start of combined treatment (AUC = 0.71).
