# Supplementary material for: Sequencing Immunotherapy and Hypofractionated Radiotherapy in Frail Patients with Locally Advanced Head and Neck Squamous Cell Carcinoma
Source: Curr Oncol. 2026 Apr 22;33(5):239. doi: 10.3390/curroncol33050239 (PMC13206541; doi:10.3390/curroncol33050239)
Supplement: Supplementary file 1 [file curroncol-33-00239-s001.zip › curroncol-4178716-supplementary.pdf]

## Supplementary Materials

**Table S1. treatment characteristics.**

| Features | Median value/number (range) |
|---|---|
| *IO administration* | |
| N° of cycles | 8 (2-32) |
| *Type of IO* | |
| Nivolumab | 7 |
| Pembrolizumab | 16 |
| *RT schedule* | |
| 40 Gy/16 fr | 16 |
| 42 Gy/14 fr | 1 |
| 35 Gy/5 fr | 1 |
| 30 Gy/5 fr | 1 |
| 25 Gy/5 fr | 1 |
| 30 Gy/10 fr | 1 |
| 50 Gy/20 fr | 1 |
| *RT technique* | |
| VMAT | 19 |
| Tomotherapy | 4 |

Abbreviation list IO: Immunotherapy; RT: Radiotherapy; VMAT: Volumetric Modulated Arc Therapy.

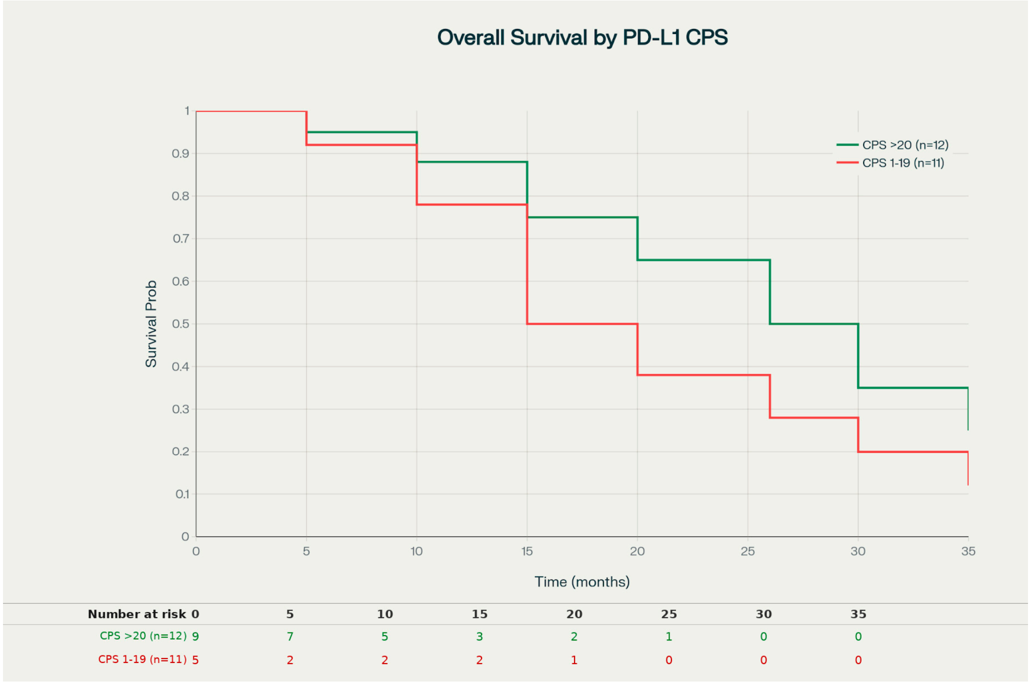


**Figure S1.** Patients with CPS >20 (group 1) demonstrated a numerically longer median OS of 26 months compared to 15 months for CPS 1–19 (group 2), although this difference did not reach statistical significance ($p = 0.9256$).

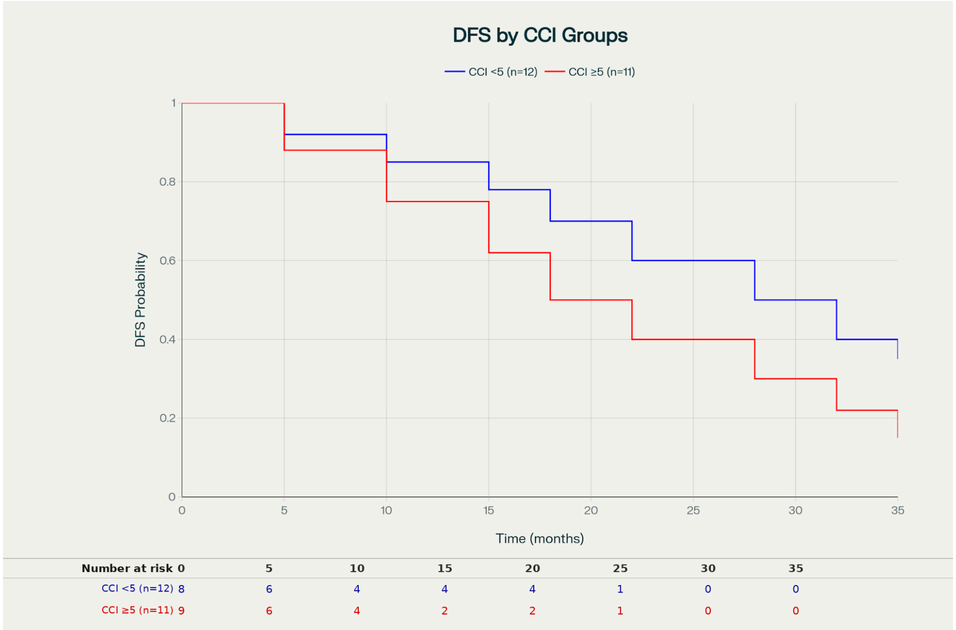


**Figure S2. Patients with CCI <5 (fewer comorbidities) demonstrated a median OS of 20 months compared to 12 months for CCI ⩾5 (p = 0.2928).**

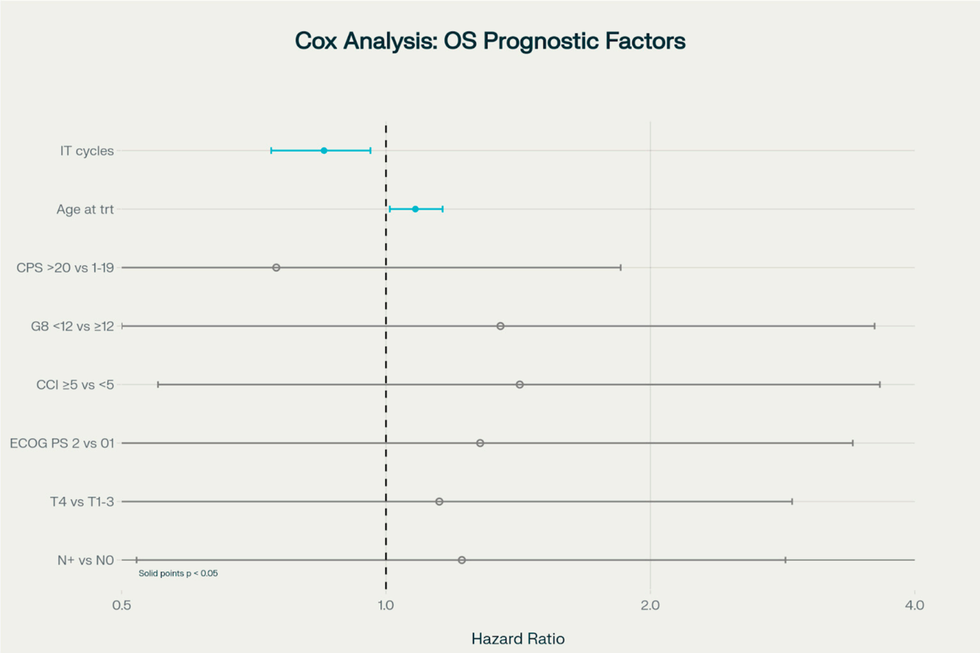


**Figure S3. Abbreviations: IT, immunotherapy; AUC, area under the curve; CPS, Combined Positive Score; G8, Geriatric 8; CCI, Charlson Comorbidity Index; ECOG PS, Eastern Cooperative Oncology Group Performance Status; NS, not significant. Blue lines and dots indicate statistically significant findings (p < 0.05).**

In the multivariate Cox proportional hazards model for OS, number of IT administrations and age at the start of combined treatment were found to be independent significant prognostic factors, while CPS score (>20 vs 1-19), G8 score (<12 vs ⩾12), CCI score (⩾5 vs <5), ECOG PS (2 vs 0-1), T stage (T4 vs T1-3) and N stage (N+ vs N0) were not significant [Figure 3]. Specifically, each additional cycle of immunotherapy was associated with improved OS, with a HR = 0.85 (95% CI: 0.74-0.96, p = 0.0189) and a higher age at the start of combined treatment was detrimental for OS, HR = 1.08 (95% CI: 1.01-1.16, p = 0.0350).

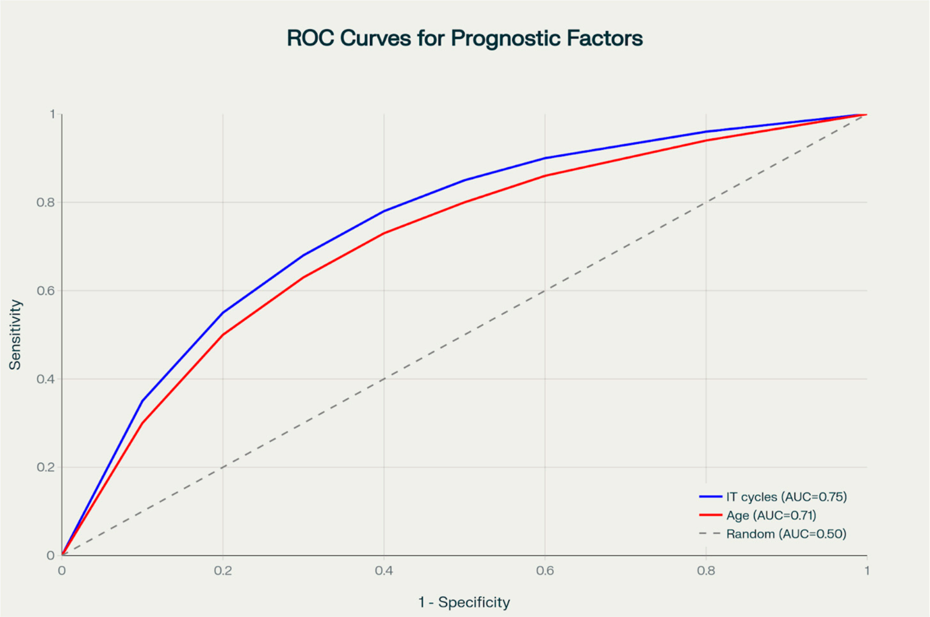


**Figure S4. ROC analysis confirmed the prognostic value of both variables: number of immunotherapy administrations (AUC = 0.75) and age at the start of combined treatment (AUC = 0.71).**

ROC analysis confirmed the prognostic value of both variables: number of immunotherapy administrations and age at the start of combined treatment (number of IT cycles: AUC = 0.75; age at treatment: AUC = 0.71)
